# Resource and technology data for spatio-temporal value chain modelling of the Great Britain energy system

**DOI:** 10.1016/j.dib.2020.105886

**Published:** 2020-06-20

**Authors:** Christopher J. Quarton, Sheila Samsatli

**Affiliations:** Department of Chemical Engineering, University of Bath, Claverton Down, Bath BA2 7AY, UK

**Keywords:** Energy system data, Technology costs, Electricity demands, Heat demands

## Abstract

A comprehensive dataset for the resources and technologies in a present-day energy system is presented. These data were used as the model input data for spatio-temporal value chain optimisation studies for the Great Britain energy system. Although focussed on Great Britain, these data may be applicable to other energy systems worldwide. Data are presented for energy resources and energy technologies. Spatio-temporal energy resource data include availabilities of primary resources such as wind and solar, and demands for heat and electricity in the domestic, commercial and industrial sectors. Economic and operating parameters for a wide range of energy technologies are presented, including resource utilisation and conversion technologies such as wind turbines and electrolysers, resource transportation such as gas transmission systems, and energy storage technologies. The data were gathered from a wide range of academic literature, technical reports, and government databases; all references are provided with the data. This dataset will be valuable for greater understanding of the energy value chain optimisation studies performed using the Value Web Model, carrying out further energy system modelling, and as a general reference for the resources and technologies in present-day energy systems.

**Specifications Table**

**Table:** 

**Subject**	Energy (General)
**Specific subject area**	Value chain optimisation of the Great Britain energy system
**Type of data**	Tables Figures
**How data were acquired**	Data were compiled from a range of sources including: • Academic publications • Technical reports • UK government databases All references are provided in this article or in the accompanying Excel file.
**Data format**	Raw (model input data)
**Parameters for data collection**	The objective was to obtain realistic estimates for present day energy resource and technology data. These data needed to be in the correct format to be used within a value chain optimisation model.
**Description of data collection**	The data were acquired through a review of literature and other references. Where possible, data were cross-checked against alternative references for reliability. In some cases, processing was carried out to obtain data in the correct format. Details of the processing (such as aggregation into the correct spatio-temporal format) are provided in this article.
**Data source location**	These data are focussed on Great Britain, but much of them are applicable worldwide (for example energy technology data).
**Data accessibility**	With the article
**Related research article**	Christopher J. Quarton and Sheila Samsatli [1] Should we inject hydrogen into gas grids? Practicalities and whole-system value chain optimisation Applied Energy [Accepted/In Press]

**Value of the Data**

• This article provides up-to-date, fully referenced spatio-temporal data for the key technologies and resources in present-day energy systems.
• These data will be valuable to modellers intending to model energy scenarios in Great Britain or elsewhere, and others looking for general references for present-day energy technologies and resources.
• This dataset provides a valuable baseline for input data to energy systems models. The data can be used for further energy systems modelling and analysis, and for cross-checking against assumptions made in other studies.
• Sharing of the data used in energy systems modelling is essential for fully understanding the model results, and for comparing the assumptions made in different studies.

## 1 Data

This article describes the full set of input data for spatio-temporal value chain optimisation of the Great Britain (GB) energy system. The data have been used in studies concerning designing value chains for multiple energy carriers (e.g. electricity, natural gas, hydrogen, syngas etc.) [2], and exploring the roles of hydrogen and inter-seasonal storage [3], carbon capture, storage and utilisation [4], and gas linepack and hydrogen injection into gas grids [1] in achieving decarbonisation targets. All of the data used in the most recent of these studies [1] can be found in the appendix of this data article, which can be downloaded in Excel format. Readers interested in the mathematical formulation of the integrated multi-vector energy value chain model, called the Value Web Model (VWM), are directed to the papers cited above.

The data in the appendix include the following:

• Spatio-temporal resource availability data, including wind speeds, solar irradiance, and availability of natural gas;
• Available land, seabed and rooftop areas for wind turbines and solar PV installations;
• Spatio-temporal electricity demands;
• Cost and operational data for a wide range of energy technologies including resource utilisation technologies (e.g. wind turbines); conversion technologies (e.g. electrolysers, gas turbines); storage technologies (e.g. overground and underground gas storage); and transportation technologies (e.g. electricity and gas transmission);
• Existing installed capacities of the technologies described above.

A detailed description of the data can be found in the appendix. The resource data are presented at the spatio-temporal aggregation used in the study. Details of the spatio-temporal format, as well as details of the processing carried out to convert data to this format, are provided in Section 2.

## 2 Experimental design, materials, and methods

### 2.1 Representation of space and time

**Fig. 1:**
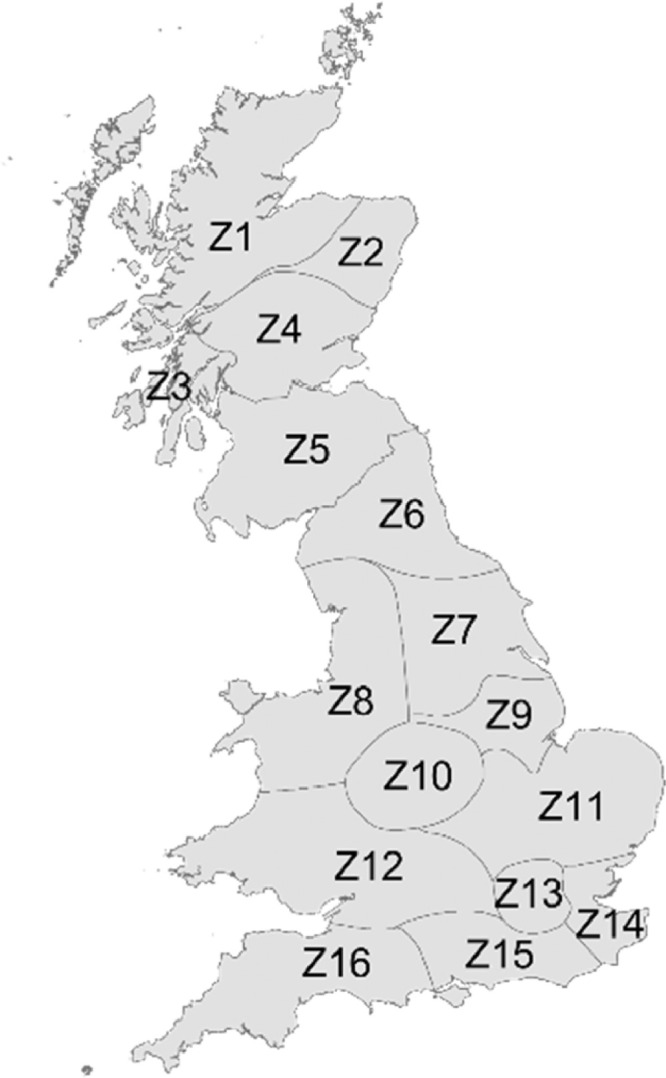
Spatial zones used to represent the Great Britain energy system. Based on the National Grid Seven Year Statement zones [5].

Spatially, the GB energy system is represented by 16 spatial zones, based on the zones used in the National Grid Seven Year Statement [5], as shown in Fig. 1.

**Fig. 2:**
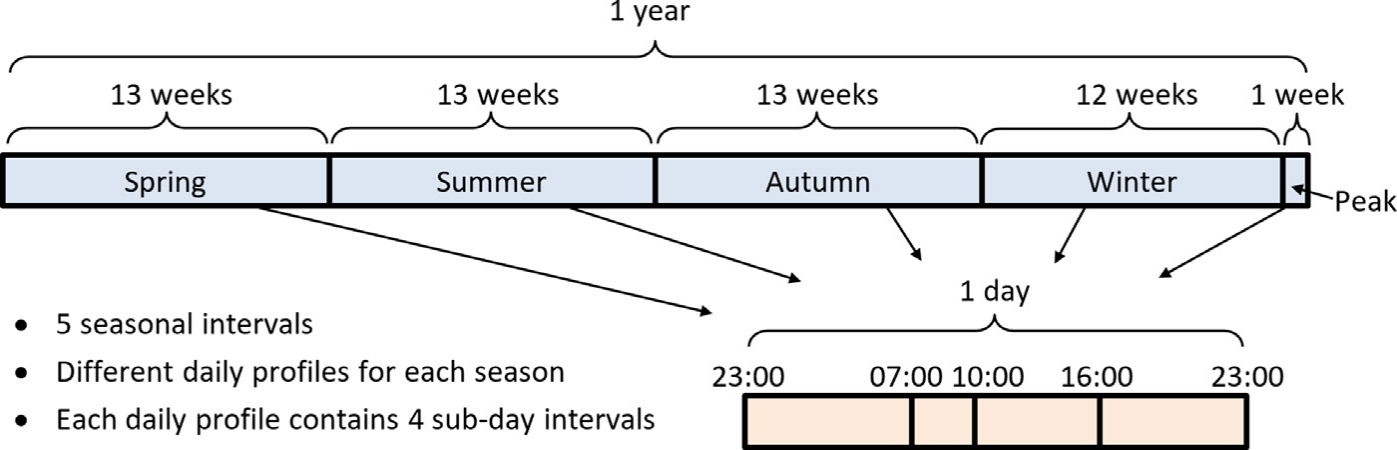
Details of the temporal aggregation.

A temporal aggregation is used in order to represent the time-varying data across the time horizon modelled. Details of the aggregation are shown in Fig. 2. Each yearly interval that is modelled is represented by five seasonal intervals, including the four main seasons and a short “peak” interval to represent energy demands on extreme occasions. Within each season, the sub-daily variation is modelled with four sub-day intervals (enabling, for example, the representation of peak energy demands in the morning and evening). Multiple yearly intervals are also modelled, and can include changes in the overall level of resource availability/demand, and changes in technology parameters (e.g. costs).

The resource data in this article have been aggregated at the spatio-temporal representation used in the optimisations using the VWM. For spatial data, either zonal average or centroidal data have been used. For temporal data, typically data have been averaged across each time interval; where alternative methods have been used, this is described in the following sections.

### 2.2 Wind speed data

Hourly wind speeds across an entire year were extracted from the Renewables Ninja database [6] for each spatial zone. Averaging wind speeds across a day or season would not represent the variability of the wind realistically, therefore “typical” wind speed profiles were generated for each season. For each zone, and for each seasonal interval, “typical” days were selected, by comparing the standard deviation of wind speed on each day to the average standard deviation across the season. The wind speeds on each typical day were then scaled to match the average wind speed across the season. Hence, an hourly profile is generated for each season that accurately represents the average wind speed for the season, and approximates the expected level of wind speed variability on a typical day. For the peak season, hourly data from winter were used, but scaled to equal the lower quartile daily wind speed for winter (rather than the average), therefore representing a possible low-wind day. Finally, data were aggregated to the model time representation by taking the average wind speed across all hours within each model hourly time interval.

This procedure was carried out for both offshore and onshore locations. The generated representative wind speeds would result in approximate average annual load factors across all zones of 24% for onshore (maximum of 31% in zone 16), and 41% for offshore (maximum of 50% for zone 1).

### 2.3 Solar resource data

The Renewables Ninja database for solar irradiance already takes into account several factors, including: solar irradiance, the sun's position, ambient temperature, the PV panel's location and orientation, and real solar farm data [7]. Data from the MERRA-2 database for 2014 were used, and a panel tilt of 40° was assumed. Hourly data from the centroid of each spatial zone across one year were extracted.

To aggregate the data to the model temporal resolution, hourly data were averaged for each season (the peak season was assumed to have the same hourly profile as the winter season), and averaged across all hours within each sub-day interval.

The data result in an average capacity factor for the country (not accounting for ancillary losses) of 13.3%. The highest capacity factor is achieved in Zone 14 (South East England), with a capacity factor of 16.0%.

### 2.4 Wind and solar existing installed capacities

Data for the existing installed capacity of wind turbines were collated from the BEIS Renewable Energy Planning Database [8]. The installed capacity of both offshore and onshore wind turbines for each spatial zone was collated based on the location information provided in the database. Only wind projects that are currently operational or under construction were included. Retirement dates were estimated based on the date upon which the project was operational, assuming a 20 year lifetime. Projects still under construction were assumed to become operational in 2020. Therefore all existing wind turbines in 2020 were assumed to have retired by 2040. Projects operational before 2005 were excluded.

As with new solar PV, existing solar installations are modelled with a representative size of 1 MW. Data for the existing installed capacity of large scale solar PV (i.e. solar farms) were collated from the BEIS Renewable Energy Planning Database [8]. Only solar installations that are currently operational or under construction were included. Retirement dates were estimated based on the date upon which the project was operational, assuming a 20 year lifetime. Projects still under construction were assumed to become operational in 2020. Therefore all existing solar installations in 2020 were assumed to have retired by 2040. Projects operational before 2005 were excluded.

The total installed capacity of rooftop solar was based on the installed capacity of solar PV receiving the feed-in tariff in the BEIS Solar photovoltaics deployment database [9]. The spatial distribution of installed rooftop solar was approximated based on a combination of the land area covered by buildings in each zone and the distribution of solar farms. Retirements of existing rooftop solar were all assumed to occur in 2040.

### 2.5 Conversion technologies installed capacities

The existing installed capacity of natural gas CCGTs was obtained from BEIS data [10]: all CCGTs commissioned since 2005 were included. For inclusion in the VWM, a representative number of the CCGT technology described in Table 11 of the Excel file (appendix) were included to give a similar power capacity per zone. No OCGTs have been commissioned since 2005, hence no existing OCGTs data were included.

Approximate data for existing natural gas heating technologies are also included, although there are limited data available for this. Only natural gas technologies are included, as these are a) predominant, and b) the most significant to the decarbonisation challenge. For (high temperature) industrial heating, it is assumed that approximately 50% of demand is currently satisfied by natural gas, based on data from [11] and the BEIS Heat Strategic Options Project [12]. Therefore, existing natural gas heating technologies are included in sufficient numbers to satisfy this demand, with half assumed to retire within one decade, and the remainder within two decades.

For domestic and commercial heating, it is assumed that the proportion of heating that is satisfied by natural gas is equal to the proportion of buildings connected to the gas grid, as provided by [13]. Hence, the number of existing technologies for domestic heating is calculated from the proportion of buildings on the gas grid and the number of households in each zone.

The number of households in each spatial zone was calculated from data from the Office for National Statistics (ONS) for 2016 [14]. For each decade, the number of households is scaled based on ONS projections for the overall number of households (England only) [15]. This supporting data are shown in Table 1.

**Table 1:** Number of households in each spatial zone (thousands) [14, 15].

Number of households (thousands)	Z1	Z2	Z3	Z4	Z5	Z6	Z7	Z8	Z9	Z10	Z11	Z12	Z13	Z14	Z15	Z16
2020	56	232	21	658	1320	1376	2460	3591	598	2909	2237	2732	56	232	21	658
2030	60	249	23	704	1413	1473	2633	3843	640	3114	2394	2923	60	249	23	704
2040	64	265	24	750	1506	1569	2805	4094	681	3317	2550	3114	64	265	24	750
2050	68	282	26	797	1599	1667	2979	4350	724	3524	2709	3309	68	282	26	797

Finally, the number of existing commercial heating technologies is calculated from the peak demand, the maximum operating rate of the gas-based technologies, and the proportion of buildings on the gas grid. Half of existing gas boilers are assumed to retire in 2030, the other half in 2040.

## Declaration of Competing Interest

The authors declare that they have no known competing financial interests or personal relationships which have, or could be perceived to have, influenced the work reported in this article.

## Appendix Supplementary materials

Image, application 1
